# Eating disorders in medical students of Karachi, Pakistan-a cross-sectional study

**DOI:** 10.1186/1756-0500-5-84

**Published:** 2012-02-01

**Authors:** Akhtar Amin Memon, Syeda Ezz-e-Rukhshan Adil, Efaza Umar Siddiqui, Syed Saad Naeem, Syed Adnan Ali, Khalid Mehmood

**Affiliations:** 1 Medical student, Dow Medical College, Dow University of Health Sciences, Karachi, Pakistan; 2 Lecturer Biostatistics, Department of Research, Dow University of Health Sciences, Karachi, Pakistan; 3 Department of Medicine, Civil Hospital, 1704/3, Federal B Area, Karachi, Pakistan

**Keywords:** Eating Disorders, EAT-26, SCOFF, Karachi

## Abstract

**Background:**

To assess the incidence of high-risk population of medical students with eating disorders in Karachi by using validated self-administered questionnaires. The earlier these disorders are diagnosed and assessed, the better the chances are for enhanced treatment and fuller recovery. Therefore, we intended to undertake a study to find out the frequency of such disorders among medical students of Karachi and design strategies to overcome them.

**Findings:**

A descriptive cross sectional study was conducted in 435 medical students of Karachi. Data was collected using 2 self administered questionnaires, the SCOFF Eating Disorders Questionnaire and the Eating Attitudes Test (EAT-26). Subjects' body mass indexes (BMI) were also calculated. The data was sorted and analyzed in SPSS version 16. According to EAT-26, 22.75% individuals were found to be at high-risk of eating disorders, with 87.9% females and 12.1% males. However, according to SCOFF questionnaire, 17% individuals were found to be at high-risk, with 78.4% females and 21.6% males. According to BMI calculation, 9% were severely underweight, 41.4% underweight, 41.1% normal, 7.6% overweight and 0.9% belonged to obese class 1.

**Conclusions:**

A significant fraction of medical students in Karachi are at high risk of development of eating disorders, females being more prone than males. Strategies should be designed to prevent occurrence of such disorders among medical students that would undoubtedly hamper the availability of dependable medical services in future.

## Background

Eating disorders refer to a group of conditions characterized by abnormal eating habits. They involve either insufficient or excessive food intake that is detrimental to an individual's physical and emotional health. Binge eating disorder, bulimia nervosa and anorexia nervosa are considered to be the most common forms of eating disorders [1]. They are among the potentially lethal psychiatric illnesses, and are predominately represented by a mental effect of preoccupation with body weight, shape and diet. In addition, eating disorders frequently occur with other psychiatric disorders such as depression, substance abuse, and anxiety disorders.

People with anorexia have an extreme fear of gaining weight, which propels them to maintain a weight far less than normal. Bulimia is characterized by a cycle of binge eating, followed by attempts to remove unwanted calories. People with binge eating disorders often eat an uncontrollable, large amount of food during the binges.

The exact cause of eating disorders is unknown. However, it is believed to be due to a combination of biological, psychological and/or environmental abnormalities. A common phrase in such conditions is that "Genetics loads the gun, environment pulls the trigger" [2].

Various studies have reported prevalence of eating disorders. In western countries, the population-based and clinical-based assessments have reported prevalence of anorexia nervosa to range from 0.1%-5.7%, while bulimia nervosa ranged from 0.3%-7.3% in female subjects [3].

A study conducted on college students reported 3.8% prevalence of bulimia nervosa in females and 0.2% in males [4]. Pyle et al. diagnosed 4.7% of female college students with eating disorders [5].

Medical students are associated with high levels of stress [6] that stands as a critically important causative factor of eating disorders. Thus, it is quite important to analyze all such instabilities in medical students who are an asset for the future of this country. Studies have been conducted in western scenario to assess eating disorders in medical students. A study from US showed that 15% of the female medical students had history of eating disorders [7].

In Pakistan, a study conducted in Lahore among 369 school girls and another study conducted in Mirpur among 271 school girls revealed one case of bulimia and no cases of anorexia, although five girls from Lahore also suffered from partial syndrome bulimia nervosa [8,9]. Another survey from Lahore among 111 volunteers showed an occurrence of two cases of bulimia nervosa and another two cases of eating disorders not otherwise specified [10].

While eating disorders are characterized as a mental health condition, they have the potential to lead to other serious physical health problems. Keeping such ominous medical consequences in view, it is naturally alarming that the future physicians of Karachi, prone to such stressful conditions might be at significantly high risk of contracting eating disorders that would hamper the availability of dependable medical services in future. The earlier these disorders are diagnosed and assessed, the better the chances are for enhanced treatment and better recovery. Therefore, we intend to undertake a descriptive study to assess the incidence of high-risk of eating disorders among medical students of Karachi.

## Methods

### Study setting and participants

This was a descriptive cross sectional study, conducted in three renowned medical colleges from Karachi namely Dow Medical College, Sindh Medical College and Aga Khan University between 1^st ^January and 30^th ^June, 2011. The study included 495 undergraduate medical students. Random sampling method was acquired. Participants from first to final year of medical school were approached directly within the college timings.

### Ethical review

The Ethical Review Board of Dow University of Health and Sciences approved the study.

### Collection of data

All participants completed a self report screening package that included the eating attitude test (EAT-26) and SCOFF questionnaire. English versions of both the questionnaires were used. No translated versions were adopted. Written consent was also obtained from all participants. Body mass index was calculated based on self reported height and weight.

The Eating attitude test-26 (EAT-26) is a validated self-administered questionnaire widely used to measure eating disorders [11]. It comprises of 26 questions for which, scoring is done on a 6-point scale from always to never. Total sum of Eat-26 scores range from zero to 78.

SCOFF is another highly accurate self-administered questionnaire widely used as a screening tool for eating disorders [12]. It comprises of 5 questions for which, scoring is done on a 2-point scale, Yes or No.

### Analysis of data

The data was entered and analyzed using the Statistical Package for the Social Sciences (SPSS) version 16. Relevant frequency and percentages were calculated for qualitative variables whereas means ± standard deviations were calculated for quantitative variables. P-values were also obtained by Pearson Chi Square Test to determine the significance of the results.

## Findings

Out of 495 individuals, 435 returned the complete questionnaires giving a response rate of 87.8%. Out of 435 individuals, 342 were female while 93 were male. According to Table 1, the mean age of the population was 20.5 years ± 1.67 years while Mean Body Mass Index Ratio (BMI) was 20.1 years ± 3.3 years. Mean age of male participants was 20.24 years ± 1.89 years, similarly it was found to be 20.65 years ± 1.60 years in females.

**Table 1:** Demographics and Mean scores of study population

		Gender	
		
		Male	Female
Age	Mean	20.24	20.65
	
	Standard Deviation	1.89	1.60

BMI	Mean	21.57	19.80
	
	Standard Deviation	4.01	3.08

Eat 26 Score	Mean	11.86	13.55
	
	Standard Deviation	9.37	9.71

SCOFF Score	Mean	1.18	1.29
	
	Standard Deviation	1.19	1.22

The Table 2 presents the descriptive for the whole data of the entire population of both the questionnaires, a difference between mean and median gives the knowledge that score are not normally distributed.

**Table 2:** Descriptive analysis of both questionnaires

	Eat 26 Score	SCOFF Score
Mean	13.19	1.27

Median	11.00	1.00

Std. Deviation	9.65	1.21

Range	75.00	5.00

The average BMI, Eat-26 Score and SCOFF score was 21.57 kg/m [2], 11.86/78 and 1.18/5 in male participants with standard deviation of 4.01, 1.18 and 1.19 respectively, similarly mean(SD) was 19.80(3.08), 13.55(9.71) and 1.29(1.22) in females respectively (Table 1).

Based on the data, two new derivatives were produced using the 75th percentile and named as the empirically derived cut-off. Hence, the cut-off value for EAT-26 was found to be more than 18, while cut-off for SCOFF was found to be more than 2.

Thus, out of 435 individuals who were screened through the two questionnaires, EAT-26 questionnaire detected 99 (22.75%) individuals with high risk of eating disorders, while SCOFF questionnaire detected 74 (17%) at high risk.

### Eating attitude test-26 (EAT-26) findings

Out of reported 99 high-risk individuals, 87 (87.9%) were females and 12 (12.1%) were males [*p *= 0.011].

Medical students of younger age group were found to be more susceptible. 65 (65.65%) were from age group 18-21, while only 34 (34.34%) were from age group 22-25 [*p *= 0.245].

Body Mass Index Ratio (BMI-ratio) of majority (n = 53, 53.5%) of the individuals screened by EAT-26 to be high-risk eating disorders fell into normal category (18.5-25 kg/m [2]). Table 3 shows the BMI results for 99 high-risk individuals [*p *= 0.002].

**Table 3:** EAT-26 results in relation to Gender, Age group and BMI ratios

	EAT-26 result		Total (n = 435)	*P*-values
			
	Positive (n = 99)	Negative (n = 336)		
**Gender:**				

Male	12 (12.9%)	81 (87.1%)	93 (100%)	0.011

Female	87 (25.4%)	255 (74.6%)	342 (100%)	

**Age group:**				

18-21 yrs	65 (21.2%)	241 (78.8%)	306 (100%)	0.245

22-25 yrs	34 (26.4%)	95 (73.6%)	129 (100%)	

**BMI:**				

Severely Underweight	09 (23.1%)	30 (76.9%)	39 (100%)	0.002

Underweight	28 (15.6%)	152 (84.4%)	180 (100%)	

Normal	53 (29.6%)	126 (70.4%)	179 (100%)	

Overweight	06 (18.2%)	27 (81.8%)	33 (100%)	

Obese Class 1	03 (75%)	01 (25%)	04 (100%)	

Out of the 99 high-risk individuals, 76.76% (n = 76) were terrified of being overweight while 68.68% (n = 68) were preoccupied with desire to be thinner. 55.56% (n = 55) were engaged in dieting behavior. However, only 9% (n = 9) vomited after eating while 73.7% (n = 73) displayed self-control around food. All the responses of questions of EAT-26 as given by positive and negative respondents have been shown in Tables 4, 5 and 6 where these questions are divided into the three Subscales of EAT-26, Dieting; Bulimia and Food Preoccupation; Oral Control respectively.

**Table 4:** Analysis of Dieting Subscale of EAT-26

Questions from Dieting Subscale of EAT-26	Frequency of response of 99 High-Risk Individuals (YES/NO)	Frequency of response of Negative 336 Individuals (YES/N0)	*P*-values
**Am terrified about being overweight**.	76/23	94/242	0.000

**Aware of the calorie content of foods that I eat**.	58/41	76/260	0.000

**Particularly avoid food with a high carbohydrate content**	39/60	21/315	0.000

**Feel extremely guilty after eating**.	36/63	5/331	0.000

**Am preoccupied with a desire to be thinner**.	68/31	75/261	0.000

**Think about burning up calories when I exercise**.	77/22	135/201	0.000

**Am preoccupied with the thought of having fat on my body**.	63/36	63/273	0.000

**Avoid foods with sugar in them**.	51/48	38/298	0.000

**Eat diet foods**.	45/54	27/309	0.000

**Feel uncomfortable after eating sweets**.	51/48	40/296	0.000

**Engage in dieting behavior**.	55/44	31/305	0.000

**Like my stomach to be empty**.	41/58	40/296	0.000

**Have the impulse to vomit after meals**.	17/82	6/330	0.000

**Table 5:** Analysis of Bulimia and Food Preoccupation Subscale of EAT-26

Questions from Bulimia and Food Preoccupation Subscale of EAT-26	Frequency of response of 99 High-Risk Individuals (YES/NO)	Frequency of response of Negative 336 Individuals (YES/N0)	*P*-values
**Find myself preoccupied with food**.	39/60	87/249	0.009

**Have gone on eating binges where I feel that I may not be able to stop**.	38/61	48/288	0.000

**Vomit after I have eaten**.	9/90	4/332	0.000

**Feel that food controls my life**.	59/40	88/248	0.000

**Give too much time and thought to food**.	47/52	37/299	0.000

**Enjoy trying new rich foods**.	28/71	107/229	0.501

**Table 6:** Analysis of Oral Control Subscale of EAT-26

Questions from Oral Control Subscale of EAT-26	Frequency of response of 99 High-Risk Individuals (YES/NO)	Frequency of response of Negative 336 Individuals (YES/N0)	*P*-values
**Avoid eating when I am hungry**.	30/69	20/316	0.000

**Cut my food into small pieces**.	75/24	116/220	0.000

**Feel that others would prefer if I ate more**.	37/62	57/279	0.000

**Other people think that I am too thin**.	40/59	111/225	0.176

**Display self-control around food**.	73/26	143/193	0.000

**Feel that others pressure me to eat**.	50/49	89/247	0.000

### SCOFF findings

Out of reported 74 high-risk individuals, 58 (78.4%) were females, 16 (21.6%) were males [*p *= 0.955]. 53 (71.6%) were from age group 18-21, while only 21 (28.4%) were from age group 22-25 [*p *= 0.792]. Thus, SCOFF reports younger age group to be more at risk.

Table 7 shows the BMI results for 74 high-risk individuals [*p *= 0.002].

**Table 7:** SCOFF results in relation to Gender, Age group and BMI ratios

	SCOFF result		Total	*P*-values
			
	Positive (n = 74)	Negative (n = 361)		
**Gender:**				

Male	16 (17.2%)	77 (82.8%)	93 (100%)	0.955

Female	58 (16.9%)	284 (83%)	342 (100%)	

**Age group:**				

18-21 yrs	53 (17.3%)	253 (82.7%)	306 (100%)	0.792

22-25 yrs	21 (16.3%)	108 (83.7%)	129 (100%)	

**BMI:**				

Severely Underweight	1 (2.6%)	38 (97.4%)	39 (100%)	0.002

Underweight	22 (12.2%)	158 (87.8%)	180 (100%)	

Normal	42 (23.5%)	137 (76.5%)	179 (100%)	

Overweight	7 (21.2%)	26 (78.8%)	33 (100%)	

Obese Class 1	2 (50%)	2 (50%)	4 (100%)	

### Logistic regression models

To find the eating disorder using EAT-26 questionnaire (Table 8), we used binary logistic backward method, in 1st step the covariate age played role insignificantly, therefore it was eliminated from the model, the final model is given as,

**Table 8:** Logistic regression model for EAT-26

		B	**S.E**.	Wald	df	**Sig**.	Odds ratio	95.0% C.I. for Odds ratio	
								
								Lower	Upper
Step 1*	Age	0.018	0.071	0.066	1	0.798	1.018	.886	1.170
	
	Gender(1)	-1.070	0.351	9.302	1	0.002	0.343	0.173	.682
	
	BMI	0.112	0.036	9.750	1	0.002	1.118	1.042	1.199
	
	Constant	-3.688	1.614	5.219	1	0.022	0.025		

Step 2*	Gender(1)	-1.077	0.349	9.501	1	0.002	0.341	0.172	0.676
	
	BMI	0.112	0.036	9.849	1	0.002	1.118	1.043	1.199
	
	Constant	-3.320	0.734	20.448	1	0.000	0.036		

a Variable(s) entered on step 1: Age, Gender, BMI

### Eat-26 disorder (Yes) = -3.320 - 1.077 (Male) + 0.112 BMI

The model explain us that, the odds of person with eating disorder was 0.341 in male as compare to female with 95% confidence interval (0.676, 0.172) and a unit change in BMI will increase the odds for disorder 0.112 time on average with 95% confidence interval (1.199, 1.043).

When the same method was performed for the SCOFF questionnaire (Table 9), we get only BMI played a significant role for eating disorder, the computed model was,

**Table 9:** Logistic regression model for SCOFF

		B	S.E.	Wald	df	Sig.	Odds ratio	95.0% C.I. for Odds ratio	
								
								Lower	Upper
Step 1*	Age	0.018	0.081	1.784	1	0.182	.898	0.767	1.052
	
	Gender(1)	-0.359	0.334	1.156	1	0.282	0.698	0.363	1.344
	
	BMI	0.161	0.039	17.223	1	0.000	1.175	1.089	1.268
	
	Constant	-2.643	1.791	2.178	1	0.140	.071		

Step 2*	Age	-0.097	0.080	1.496	1	0.221	0.907	0.776	1.060
	
	BMI	0.150	0.037	16.274	1	0.000	1.162	1.080	1.249
	
	Constant	-2.697	1.777	2.303	1	0.129	0.067		

Step 3*	BMI	0.148	0.037	15.959	1	0.000	1.160	1.078	1.247
	
	Constant	-4.657	0.799	34.011	1	0.000	0.009		

a Variable(s) entered on step 1: Age, Gender, BMI

### Scoff eating disorder (Yes) = -4.657 + 0.148 BMI

Explain us that, the unit change in BMI will increase the odds for disorder 0.148 times on average, with 95% confidence interval (1.247, 1.078).

## Discussion

Our study reports that significant proportion of medical undergraduates are at high risk to suffer from eating disorders with 99 (22.75%) individuals scoring above the threshold for EAT-26 questionnaire while 74 (17%) scoring above the threshold for SCOFF questionnaire. This is much more than as compared to recently reported eating disorder symptoms in 9.59% among Latino college students by Reyes-Rodriguez et al. [13]. This strengthens the fact that eating disorders are a current mounting concern in our region in relation to other parts of the world.

We reported a significant majority of females being at high-risk of eating disorders as compared to males. Pope et al. reported a similar ratio between male and female students wherein a high proportion of female subjects (anorexia = 1% to 4.2% or bulimia = 6.5% to 18.6%) suffered from eating disorders while none of the male subjects was reported positive [14]. Another study reported females (binge eating, n = 49% bulimia n = 4%) at a greater risk to develop eating Disorders [15].

As also seen in our study with universities located in urbanized locations, females in such settings, as an avid media followers, are particularly more prone for developing eating disorders. Various studies in different settings have highlighted the role of media exposure and its psychological effect particularly on females with resultant development of body dissatisfaction culminating in eating disorders [12,16]. In a setting like Pakistan, the increasing drive of particularly females to emulate European culture as viewed via media has led to an unhealthy stringent dieting and exercising regime [17].

Interestingly, our study showed that of the individuals with normal BMI values, 29.6% still suffered from eating disorders as diagnosed by EAT-26 (n = 53/179, Table 3) while according to SCOFF, 23.5% (42/179, Table 7) of normal individuals were suffering from eating disorders.

Overweight individuals were found to be more likely to have eating disorders in relation to underweight individuals. According to SCOFF, 21.2% (7/33) of the overweight while 12.2% (22/180) of underweight individuals scored above the cutoff score thus were likely to have eating disorders. Similarly in accordance to EAT-26, 18.2% (06/33) of the overweight individuals while 15.6% (28/180) of the underweight individuals were likely to have eating disorders.

The plausible explanation for our finding that underweight individuals are less likely to have eating disorders in relation to overweight individuals can be provided by elucidating that eating disorders are morbidities with psychological basis, even individuals with normal body mass index can have likelihood of these disorders. Thus, it depends, only in part, on the actual body mass. One debatable reason can be the psychological satisfaction attained after achieving the desired body shape. Thus, the causative factor that propelled them to have eating disorders was now resolved. Further, studies must be carried out in future to find out if eating disorders resolve after a person achieves the desired lean body image. Other possible reasons for low weight individuals can be the genetic factors, as well as undernutrition. Pappas et al. also reported a high rate of undernutrition in Pakistani population [18].

Also for each particular class of BMI, SCOFF diagnosed more than twice individuals as likely to have eating disorders as compared to EAT-26. This can be seen clearly in normal, underweight and overweight individuals.

Eating disorders particularly anorexia nervosa is reported to derange several system with resultant complications ranging from purpura, liver dysfunction, osteoporosis, diabetic complications to acrocyanosis. Particularly, anorectic patients have been reported to die at a premature age possibly from one of the above stated medical complications [19]. This disconcerting information should come in the knowledge of such individuals suffering from the disorder or at a high risk of developing one, who involve grossly in unhealthy dieting or purging cycle, particularly females.

The limitations of our study includes that we have focused only on medicals students in colleges from urban set up. Further studies should be carried out which check the pattern of eating disorders in rural set up. Furthermore, the most important limitation is the furtiveness and disagreement, attributed to many of the subjects suffering from eating disorders. This is common in almost all the studies on eating disorders.

Further surveys needs to be conducted that will co-relate socio economic group, ethnicity and relationship status (divorced married or single) with the development of the disorder which in other studies have been reported to be highly associated with the types of eating disorders. Early detection of such factors causing eating disorders is important as having a significant impact in treatment of such disorders at an early stage with resultant greater efficiency of performance by Future physicians [20].

## Competing interests

The authors declare that they have no competing interests.

## Authors' contributions

M K gave the idea of the study. A SE designed the study. A SE and M AA prepared the synopsis for IRB approval. M K reviewed and edited the synopsis. A SE, M AA, S EU, N SS collected the data. M AA and S EU entered the data on SPSS. A SA, M AA and N SS analyzed the data on SPSS. A SE, M AA, S EU, N SS wrote the manuscript. M K review and edited the manuscript. All authors read and approved the final manuscript.
